# Characterization of Polyphenolic Content in the Aquatic Plants *Ruppia cirrhosa* and *Ruppia maritima* —A Source of Nutritional Natural Products

**DOI:** 10.3390/molecules23010016

**Published:** 2017-12-22

**Authors:** Kjersti Hasle Enerstvedt, Anders Lundberg, Monica Jordheim

**Affiliations:** 1 Department of Chemistry, University of Bergen, Allégt. 41, N-5007 Bergen, Norway; Kjersti.Enerstvedt@uib.no; 2 Department of Geography, University of Bergen, Fosswinckelsft. 6, N-5020 Bergen, Norway; Anders.lundberg@uib.no

**Keywords:** *Ruppiaceae*, chicoric acid, flavonoids, NMR characterization, quantification, antioxidant assay

## Abstract

Herein, the polyphenolic content in extracts of *Ruppia cirrhosa* (Petagna) Grande and *Ruppia maritima* L.was fully characterized for the first time. High amounts of the main compound chicoric acid (**CA**) (≤30.2 ± 4.3 mg/g) were found in both *Ruppia* species. In addition, eight flavonoids, namely the 3-*O*-glucopyranosides and 3-*O*-galactopyranosides, as well as malonylated 3-*O*-glycosides of quercetin and isorhamnetin, were isolated and identified. The antioxidant activity of *Ruppia cirrhosa* extracts and isolated compounds was investigated spectrophotometrically by a 1,1-diphenyl-2-picrylhydrazyl (DPPH·) radical scavenging assay. IC_50_ values were 31.8–175.7 μg/mL for *Ruppia cirrhosa* extracts and 12.1–88.4 μg/mL for isolated flavonoids. Both individual and total phenolic and flavonoid content were quantified in crude extracts using analytical HPLC. The relative high amount of total flavonoids ranged from 5.9 to 14.7 mg/g in both species, with concentrations of individual flavonoids ranging from 0.4 to 2.9 mg/g dry weight. The content of chicoric acid was twofold more in *Ruppia maritima* than in *Ruppia cirrhosa*. Seasonal variation of the quantitative content in *Ruppia cirrhosa* was examined. Total flavonoid content ranged from 8.4 mg/g in October to 14.7 mg/g in August, whereas the highest concentration of chicoric acid was observed in March (29.2 mg/g).

## 1. Introduction

The marine environment is a potential source for a wide variety of nutritional natural products. Seaweeds are used as human food or as raw materials for the production of compounds of nutritional interest [1]. On the other hand, marine angiosperms, such as seagrasses, are known for their content of secondary metabolites [2,3]; however, these are very little exploited to find commercially valuable natural products. A few seagrass species, especially of the genus *Zostera*, *Halophila*, *Posidonia*, *Thalassia* and *Syringodium*, have been investigated for their content of phenolics and flavonoids [3,4,5,6,7,8,9,10,11,12,13]. 

The widgeon grass family (*Ruppiaceae*) is a submersed aquatic angiosperm widely distributed in temperate and tropical regions all over the world. *Ruppia* species usually occur in brackish or saline waters, but can also be found in diluted fresh water or fresh water with high salinity, and only rarely under marine conditions [14,15,16]. In Norwegian coastal waters, two *Ruppia* species have been found, namely *Ruppia maritima* L. and *Ruppia cirrhosa* (Petagna) Grande, the latter occasionally synonymized under *R. spiralis* L. ex Dumort. Both species can be found in single populations with no other vascular plants present, and they are hardly ever found together. *R. maritima* can sometimes be found in proximity of *Zostera noltii* populations, while *R. cirrhosa* can be found with or close to *Zostera marina* L. populations.

The number of studies investigating secondary metabolites in *Ruppia* species are limited, and a full analysis of polyphenolic content is lacking [7,10,17]. In 1973 Boutard et al. [7] analyzed and identified two flavonoids in *R*. *maritima* based on chrysoeriol and possibly luteolin. Harborne and Williams reported in 1976 an unidentified glycosylflavone, as well as three caffeoyl conjugates in *R. maritima*, whereas no phenolic derivatives were found in *R*. *cirrhosa* [10]. Haynes and Roberts indicated later the presence of flavonols in one *Ruppia* species [17], yet these results remain unpublished, and no accurate identification of the flavonols has been concluded. The previous identification work is based on TLC retention times and electrophoretic surveys [7,10]. 

The aim of this work was to characterize the phenolic content of *R. cirrhosa* and *R*. *maritima* collected from Norwegian coastal waters with the aims of finding a new source of nutritional natural products. To our knowledge, this is the first report on complete structural characterization of both flavonoids and one phenolic acid in these two species and our quantitative studies revealed high amounts of the potent chicoric acid (**CA**) [18].

## 2. Results and Discussion

### 2.1. Characterization of Polyphenolic Compounds in Ruppia cirrhosa

**Figure 1:**
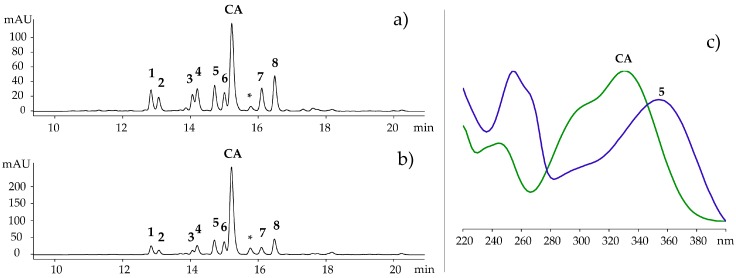
(**a**–**c**) HPLC chromatogram of *Ruppia cirrhosa* (**a**) and *Ruppia maritima* (**b**) recorded at 360 ± 10 nm; (**c**) UV-Vis spectrum of isorhamnetin 3-*O-β-*d-galactopyranoside (**5**) and chicoric acid (**CA**). See Figure 2 for structures, **1**–**8** and **CA**. * unidentified caffeoyl unit.

**Figure 2:**
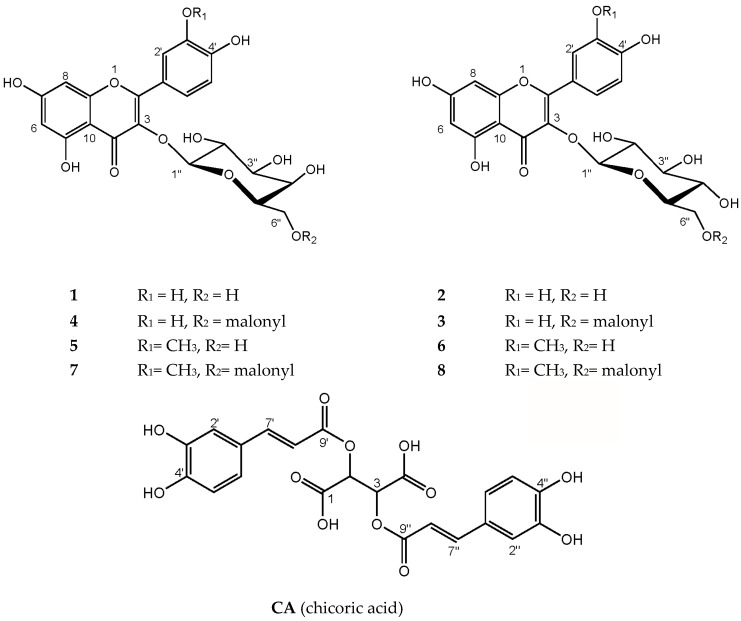
Structures of the main phenolic compounds found in *Ruppia cirrhosa* and *Ruppia maritima*. **1** = quercetin 3-*O*-*β*-d-galactopyranoside, **2** = quercetin 3-*O-β-*d-glucopyranoisde, **3** = quercetin 3-*O*-*β*-d-(6″-*O*-malonyl)glucopyranoside, **4** = quercetin 3-*O-β-*d-(6″-*O*-malonyl)galactopyranoside, **5** = isorhamnetin 3-*O-β-*d-galactopyranoside, **6** = isorhamnetin 3-*O-β-*d-glucopyranoside, **7** = isorhamnetin 3-*O-β-*d-(6″-*O*-malonyl)galactopyranoside, **8** = isorhamnetin 3-*O*-*β*-d-(6″-*O*-malonyl)-glucopyranoside, **CA** = chicoric acid.

The HPLC profile (Figure 1) of the crude extract of *R. cirrhosa* detected at 360 ± 10 nm revealed one phenolic acid and eight flavonoids (Figure 2). After purification of the concentrated extract by Amberlite XAD-7 (Sigma-Aldrich, St. Louis, MO, USA) chromatography, the compounds were isolated by preparative HPLC and analyzed using high resolution LC‒MS and 1D and 2D NMR spectroscopy. Their physiochemical and spectral data were compared to previously reported values in literature, and the compounds were identified as quercetin 3-*O-β-*d-galactopyranoside (**1**) [19,20,21], quercetin 3-*O-β-*d-glucopyranoside (**2**) [19,21,22], quercetin 3-*O-β-*d-(6″-*O*-malonyl)galactopyranoside (**4**) [23], isorhamnetin 3-*O-β-*d-galactopyranoside (**5**) [24,25], isorhamnetin 3-*O-β-*d-glucopyranoside (**6**) [22,25,26], isorhamnetin 3-*O-β-*d-(6″-*O*-malonyl)galactopyranoside (**7**) [23,27], isorhamnetin 3-*O-β-*d-(6″-*O*-malonyl)-glucopyranoside (**8**) [27] and chicoric acid (**CA**) [28]. Quercetin 3-*O-β-*d-(6″-*O*-malonyl)-glucopyranoside (**3**) was identified by comparison with an analytical standard (≥85% (HPLC), Sigma-Aldrich).

The main phenolic acid in both *Ruppia* species was chicoric acid (**CA**), which has been found previously in the seagrasses *Cymodocea nodosa* U. [29], *Syringodium filiforme* K [12], *Posidionia oceanica* L. [30,31,32] and *Thalassia hemprichii* (Ehrenb.) Ash. [33]. This is the first time flavonoids **1**–**8** and chicoric acid have been identified in *R. cirrhosa* and *R. maritima*. The flavonoids quercetin 3-*O-β-*d-glucopyranoside and isorhamnetin 3-*O-β-*d-glucopyranoside have previously been identified in the seagrass *C. nodosa* [29]. As far as we know, this is the first report of 3-*O*-galactopyranosides and malonylated glycosides of quercetin and isorhamnetin in aquatic plants.

### 2.2. DPPH Radical Scavenging of Ruppia Polyphenols

DPPH is a stable free radical with a maximum absorbance at 517 nm (deep purple colour). When reacting with a radical scavenger it donates a hydrogen and acquires a colorless reduced form. The loss of purple colour correlates with scavenging activity of the compound, and IC_50_ values are commonly used to determine the compounds ability to scavenge radicals. The IC_50_ values of *R. cirrhosa* extracts and isolated compounds are shown in Table 1. Due to insufficient amounts of sample material, DPPH· scavenging activity of *R. maritima* was not tested. The *R. cirrhosa* extract exhibited an IC_50_ value of 152.9–175.7 μg/mL, which is considered low to moderate radical scavenging activity [34]. These results are comparable to antioxidant activities of crude extracts of the seagrasses *Halodule ovalis* (IC_50_ 130 μg/mL) [35], *Syringodium isoetifolium* (IC_50_ 96.34 μg/mL), *Enhalus acoroides* (IC_50_ 115.79 μg/mL), *Cymodocea rotundata* (IC_50_ 123.72 μg/mL) and *Thalassia hemprichii* (IC_50_ 214.68 μg/mL) [36]. However, after partition with ethyl acetate, the aqueous phase of *R. cirrhosa* exhibited very strong radical scavenging activity, with an IC_50_ value of 31.8 ± 3.2 μg/mL. To our knowledge, this is the first reported results on DPPH· scavenging activity of *R. cirrhosa* extracts.

**Figure 3:**
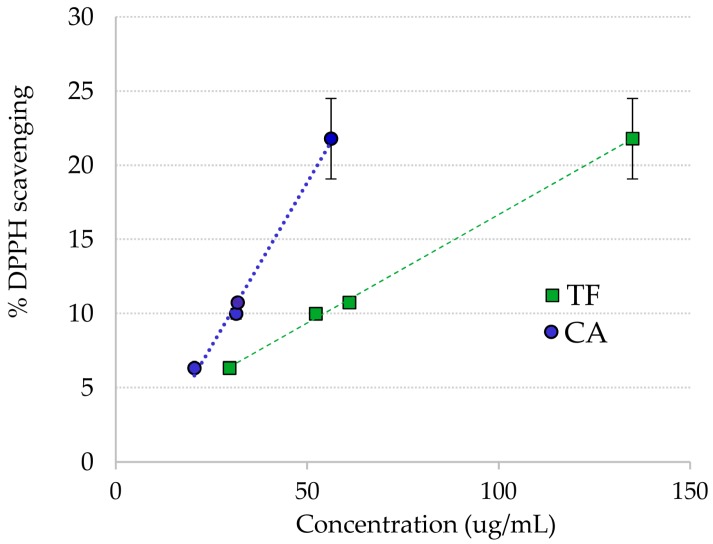
DPPH· radical scavenging vs. concentration of chicoric acid (**CA**) and total flavonoids (TF) in *Ruppia cirrhosa* crude extracts.

The extract from the plant material collected in October had a slightly lower scavenging activity than the *R. cirrhosa* extract from August. This may be related to the lower phenolic content found (Table 4). In addition, the percent scavenging of four crude extracts of *R. cirrhosa* with known concentrations of both flavonoids and chicoric acid was examined (Figure 3), revealing a correlation between antioxidant scavenging and concentration of total flavonoids and chicoric acid.

The individual flavonoids were isolated in pairs on preparative HPLC. DPPH· radical scavenging assays were performed to test the antioxidant activities of the flavonoids. The IC_50_ values of the isolated flavonoids and reference compounds are shown in Table 1 and Table 2, respectively.

Purified mixture of quercetin 3-*O-β-*d-(6″-*O*-malonyl)glucopyranoside (**3**) and quercetin 3-*O-β-*d-(6″-*O*-malonyl)galactopyranoside (**4**) showed very strong antioxidant activity, with an IC_50_ value of 12.1 ± 3.3 μg/mL. The measured value is similar to the IC_50_ values obtained for the reference standards quercetin (5.5 ± 0.3 μg/mL), quercetin 3-*O-β-*d-glucopyranoside (11.0 ± 1.0 μg/mL) and rutin (13.9 ± 0.7 μg/mL), once molar mass is accounted for. Flavonoids with an isorhamnetin aglycone (compounds **5**–**8**) showed lower antioxidant activity than the quercetin-based flavonoids (**3** and **4**), explained by the number of free hydroxyl groups on the aglycone B-ring [37]. Interestingly, the malonylated isorhamnetin *O*-glycosides **7** and **8** showed much higher antioxidant activity than the corresponding isorhamnetin *O*-glycosides **5** and **6**, with IC_50_ values of 51.7 ± 6.8 μg/mL and 88.4 ± 7.0 μg/mL, respectively.

DPPH· scavenging with chicoric acid (**CA**), isolated from *R. cirrhosa*, resulted in a higher IC_50_ value (23.0 ± 3.2 μg/mL) than the one seen for the mixture of quercetin 3-*O-β-*d-(6″-*O*-malonyl)glucopyranoside (**3**) and quercetin 3-*O-β-*d-(6″-*O*-malonyl)galactopyranoside (**4**). Compared to the isolated isorhamnetin-based flavonoids (**5** & **6** and **7** & **8**) however, **CA** showed stronger scavenging and lower IC_50_ value. The chicoric acid (**CA**) isolated in this study had a higher IC_50_ value (23.0 ± 3.2 μg/mL) (Table 1) than the one measured for the reference compound (9.7 ± 1.7 μg/mL) (Table 2). Since DPPH is a highly concentration sensitive method, variations in IC_50_ values for the same compound is often seen [38,39,40,41,42,43,44,45]. No significant impurities were observed for the isolated sample of **CA** in the present study using HPLC and NMR for purity determination. However, water content, especially if the compound is hygroscopic, and inorganic salt content will normally not be determined by these methods [46]. Nonetheless, both the isolated **CA** and reference compound showed very strong antioxidant activity.

### 2.3. Quantitative Analysis of Polyphenolic Content in Ruppia 

**Figure 4:**
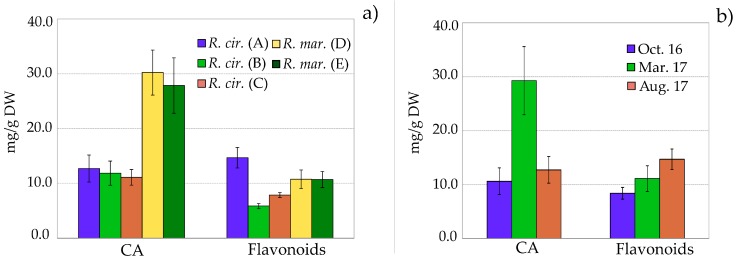
(**a**) Flavonoid and chicoric acid (**CA**) content in leaves of *Ruppia cirrhosa* (*R. cirr.*) and *Ruppia maritima* (*R. mar*.) collected from different localities; (**b**) Flavonoid and chicoric acid (**CA**) content in leaves of *Ruppia cirrhosa* collected in October 2016, March 2017 and August 2017. Amounts are expressed in mg/g (mean value ± SD, *n* = 4) dry weight, based on quercetin 3-*O*-*β*-d-glucopyranoside (flavonoids) or caffeic acid (**CA**) equivalents.

The quantitative content of individual flavonoids **1**–**8** and chicoric acid was characterized in three *R. cirrhosa* and two *R. maritima* populations, collected from different localities at the east and west coast of Norway (A–E) (Table 3). As seen in Figure 4a, the flavonoid content was significantly higher in *R. cirrhosa* from the Bergen location (A) compared to the other *R. cirrhosa* populations from the west coast of Norway (B and C). 

No significant differences in the total flavonoid or phenolic content of the two *R. maritima* populations from the east coast were observed (D and E). However, significant differences in the distribution of the individual flavonoids were seen. The *R. maritima* samples from Tønsberg (D) showed a higher content of the quercetin *O*-glycosides **1** and **2**, whereas *R. maritima* samples from the Råde (E) location contained higher amounts of the malonylated isorhamnetin *O*-glycosides (**7**) and (**8**). 

The total flavonoid content was 5.9–14.7 mg/g (DW) for *R. cirrhosa* and 10.7 mg/g (DW) for *R. maritima*, respectively (Table 3). These amounts are in the same scale as the amounts reported for common edible flavonoid sources such as *Allium* (≤5.08 mg/g DW), cranberry (2.15 mg/g DW) and dried oregano (15.46 mg/g DW) [47,48,49,50]. In marine European seagrass species as *Zostera marina* and *Zostera noltii* flavonoid amounts in the range of 13.5–24.5 mg/g (DW) and 3.38–34.3 mg/g (DW) have been found, respectively [9,51].

The concentrations of chicoric acid (**CA**) were significantly higher in *R. maritima* (30.2 and 27.9 mg/g) than in *R. cirrhosa* (11.1–12.7 mg/g). It seems natural to conclude that *R. maritima* generally have a higher production of **CA** although, although it should be taken into consideration that the *R. maritima* samples were collected from a different part of Norway. Differences in chicoric acid accumulation may be a function of nutritional and/or environmental stress, but there is a need for more research on how chicoric acid accumulation in plants is regulated [18]. In leaves of *Cymodocea nodosa* and *Syringodium filiforme*, the amounts of chicoric acid have been reported to range from 8.13–27.4 mg/g and 0.94–5.26 mg/g, respectively [12,29]. Chicoric acid has also been found in *Posidionia oceania* from the Mediterranean Sea, however, the quantitative content varied greatly. The maximum content of chicoric acid was 0.1386 mg/g in young leaves of *P. oceanica* collected in the Aegean sea outside Turkey, whereas both detrital and fresh leaves of *P. oceanica* from four different localities in the western part of the Mediterranean sea were found to contain up to 12.78 mg/g chicoric acid [31,32]. The high level of **CA** (≤30.2 ± 4.3 mg) found in this study is comparable to the content of **CA** in the known source *Echinacea purpura* [52,53,54], proposing *Ruppia* to be a new and valuable source of chicoric acid (**CA**). Chicoric acid is high value-added on the nutraceutical market, due to its possible health benefits and its relative rare occurrence in the plant kingdom [12,18].

Fluctuations in natural product concentrations should be taken into consideration before scheduling harvest dates or planning herbal product manufacturing [18]. In order to get an impression of the seasonal fluctuations of phenolics in *Ruppia*, the total flavonoid and **CA** content in *R. cirrhosa* collected from the Bergen location (A) in October, March and August were analyzed (Table 4, Figure 4b). During the winter season (December-February) the biomass on the examined locality was scarce. 

The concentration of flavonoids in *R. cirrhosa* was significantly higher in August (14.7 ± 1.9 mg) compared to October (8.4 ± 1.1 mg) and March (11.1± 2.4 mg). The concentration of **CA** in *R. cirrhosa* measured in March (29.2 ± 6.3 mg) was over twice the amounts found in August (12.7 ± 2.5) and October (10.6 ± 2.5). The observed seasonal variation of flavonoids and phenolic acid indicates a similar pattern as we have previously seen in *Zostera marina* [51], with higher concentrations in spring and summer. These trends are associated with environmental stress factors, mainly UV radiation—as seen for terrestrial plants [55,56]. It is also likely that because the young leaves are still growing, they are consequently more vulnerable for microbial/fungal and herbivory attacks, which will result in an increased production of phenolics [57]. Yet, to achieve more accurate and reliable data on the seasonal variation in relation to environmental factors, a more comprehensive study of the content of both flavonoids and chicoric acid in *R. maritima* and *R. cirrhosa* is needed.

## 3. Experimental

### 3.1. General Instrumentation

#### 3.1.1. Analytical HPLC

Agilent 1100 HPLC system (Agilent Technologies, Santa Clara, CA, USA) equipped with a HP 1050 diode array detector and a 200 × 4.6 mm inside diameter, 5 μm ODS Hypersil column (Supelco, Bellefonte, PA, USA). Two solvents, (A) water (0.5% TFA) and (B) acetonitrile (0.5% TFA), were used for elution. The elution profile for HPLC consisted of initial conditions with 90% A and 10% B followed by a linear gradient elution to 50% B. The flow rate was 1.0 mL/min, and aliquots of 15 μL were injected with an Agilent 1100 series microautosampler. The UV-Vis absorption spectra were recorded online during HPLC analysis over the wavelength range of 240–600 nm in steps of 2 nm. 

#### 3.1.2. Preparative HPLC

The preparative HPLC system used a Gilson 321 pump (Gilson S. A., Villiers-le-Bel, France), equipped with an Ultimate 3000 variable wavelength detector (Dionex, Thermo Fisher Scientific, Sunnyvale, CA, USA), a 25 × 2.12 cm (10 μm) UniverSil C18 column (Fortis Technologies Ltd., Neston, UK), and the solvents (A) water (0.1% formic acid) and (B) acetonitrile (0.1% formic acid). The elution profile for HPLC consisted of initial conditions with 90% A and 10% B followed by isocratic elution for the next 5 min, and the subsequent linear gradient conditions: 5–18 min (to 16% B), 18–22 min (to 18% B), 22–26 min (to 23% B), 26–31 min (to 28% B), and 31–32 min (to 40% B), with isocratic elution at 32–40 min (40% B) and a final linear gradient elution at 40–43 (to 10% B). The flow rate was 15 mL/min, and aliquots of 800 μL were injected. 

#### 3.1.3. LC–MS

High-resolution LC-electrospray mass spectrometry (HR‒LCMS) (ESI+/TOF), spectra were recorded using a AccuTOF JMS-T100LC (JEOL, Peabody, USA) in combination with an Agilent Technologies 1200 Series HPLC system at the following instrumental settings/conditions; Ionization mode: positive, ion source temperature = 250 °C, needle voltage = 2000 V, desolvation gas flow = 2.0 L/min, nebulizing gas flow = 1.0 L/min, orifice1 temperature = 100 °C, orifice2 voltage = 6 V, ring lens voltage = 18 V, ion guide peak voltage = 2000 V, detector voltage = 2300 V, acquisition range = 100–1000 *m*/*z*, spectral recording interval = 0.5 s, wait time = 0.03 ns and data sampling interval = 0.5 ns. The sample was dissolved in a mixture of water and acetonitrile with 0.1% formic acid. The elution profile for HPLC consisted of initial conditions with 90% A (water with 0.1% formic acid) and 10% B (acetonitrile with 0.1% formic acid), isocratic elution 0–2 min, followed by a linear gradient elution to 50% B (2–15 min). A 50 × 4.6 mm internal diameter, 1.8 μm Agilent Zorbax Eclipse XDB C18 column was used for separation.

#### 3.1.4. NMR-Spectroscopy

One-dimensional ^1^H and ^13^C distortionless enhancement by polarization transfer (DEPT-135), two-dimensional heteronuclear single quantum coherence (^1^H-^13^C HSQC), heteronuclear multiple bond correlation (^1^H-^13^C HMBC), heteronuclear 2 bond correlation (^1^H-^13^C H2BC), double quantum filtered correlation (^1^H-^1^H DQF COSY), heteronuclear single quantum coherence-total correlation spectroscopy (^1^H-^13^C HSQC-TOCSY), homonuclear *J*-resolved (^1^H *J*-RES) and total correlation spectroscopy (^1^H-^1^H TOCSY) experiments were obtained on a Bruker 850 MHz instrument (Bruker BioSpin, Zürich, Switzerland) equipped with a cryogenic probe. The spectral widths were 10–15 ppm and 165–220 ppm for the ^1^H and ^13^C-dimensions, respectively. The number of collected data points was 2048 for ^1^H-dimension in most 2D experiment (4096 in HMBC), and 256 in the ^13^C dimension. The 2D experiments HMBC, HSQC and H2BC were acquired with non-uniform sampling (NUS = 20–50%). The coupling constants were 145 Hz for ^1^*J*_CH_, 8 Hz for long range couplings (HMBC) and 120–160 Hz for ^2^*J*_CH_ (H2BC). Recycle delay was 2 s in all experiments. Sample temperatures were stabilized at 298 K. The deuteriomethyl ^13^C signal and the residual ^1^H signal of the solvent (*d*_6_-DMSO or *d*_4_-MeOD) were used as secondary references (*δ* 39.5/2.5 and 49.1/3.31 from TMS, respectively).

### 3.2. Plant Material and Study Sites

Samples of *R. cirrhosa* and *R. maritima* were collected during spring low tide by hand from five different study sites in the southern coast of Norway: Bergen, Røytepøyla (A) (60°15′34.5″ N, 05°15′57.9″ E), Etne, Gjersvik, (B) (59°38′41.5″ N, 05°55′18.8″ E), Tysvær, Hadleholmen (C) (59°23′44.1″ N, 05°28′29.6″ E), Tønsberg, Bliksekilen (D) 59°19′25.7″ N, 10°29′58.2″ E) and Råde, Skjeløy (E) (59°17′00.4″ N, 10°44′33.5″ E). Voucher specimen of *Ruppia cirrhosa* and *Ruppia maritima* have been deposited in the Herbarium BG (Voucher BG/S 164805 and 53439) at the University Museum of Bergen, Bergen. Plant identification was based on plant morphology and habitat ecology. Leaves of both species are brown-greenish, narrowly linear, sheathering at the base, and fine teethed at the apex. Sheaths of *R. maritima* are slightly inflated; sheaths of *R. cirrhosa* are typically conspicuously inflated. Flowers of both species are hermaphroditic and small, in two-flowered, pedunculate spikes. Perianth is absent. Peduncles in *R. cirrhosa* are 8–15 cm long, sometimes longer, and spirally coiled when fruits are mature. Peduncles in *R. maritima* are shorter; 4–6 cm long, often somewhat recurved in fruit but never spirally coiled. *R. cirrhosa* is typically 30–50 cm long, whereas *R. maritima* often is 10–15 cm long, sometimes up to 30 cm long. *R. maritima* is found mostly in the hydrolittoral zone, sometimes down to the upper part of the sublittoral zone, growing at ±0.5 m deep, whereas *R. cirrhosa* occurs in the sublittoral zone and is permanently submerged at depths of 0.5–1.5 m. Both species are found on soft substrata, such as mud and silt. *R. maritima* is also found on fine sand. 

### 3.3. Extraction, Purification and Identification 

The collected plant material was washed thoroughly in fresh water and air-dried. The root was separated from the rest of the plant, and the material was cut in small pieces and stored at −20 °C, when not used. Air-dried leaves of *R. cirrhosa* were extracted with 50% aqueous methanol (HPLC) for 24 h at room temperature. The extraction was repeated 4 times. The combined extracts were filtered through glass wool, and the volume was further reduced using a rotavapor. The concentrated aqueous extract was partitioned against ethyl acetate three times. The content of both the ethyl acetate and water phase was examined on HPLC. About a third of the aqueous extract was applied to an Amberlite XAD-7 column (5 × 20 cm), and eluted with distilled water until no colour was observed, then methanol was applied. Collected fractions were analyzed on analytical HPLC and concentrated using a rotavapor. The semi-purified plant extract was submitted to preparative HPLC to obtain purified compounds. The physiochemical and spectral data of the flavonoids and chicoric acid were as follows:

*Quercetin 3-O-β-d-galactopyranoside* (**1**): Yellow amorphous powder (MeOH); UV/Vis *λ*_max_ nm 353, 256, 264 (sh); HRLC-MS *m*/*z* 465.1015 [M + H]^+^, ^1^H-NMR (*d*_6_-DMSO, 850.13 MHz) *δ* (ppm), aglycone; 7.66 (1H, dd, *J* = 8.6, 2.3 Hz, H-6′), 7.53 (1H, d, *J* = 2.3 Hz, H-2′), 6.82 (1H, d, *J* = 8.4 Hz, H-5′), 6.41 (1H, d, *J* = 1.9 Hz, H-8), 6.20 (1H, d, *J* = 1.9 Hz, H-6), sugar; 5.37 (1H, d, *J* = 7.7 Hz, H-1″), 3.56 (1H, m, H-2″), 3.37 (1H, dd, *J* = 9.6, 3.6 Hz, H-3″), 3.65 (1H, m, H-4″), 3.33 (1H, m, H-5″), 3.29 (1H, dd, *J* = 10.8, 5.8 Hz, H-6a″), 3.46 (1H, dd, *J* =10.8, 6.2 Hz, H-6b″). ^13^C-NMR (*d*_6_-DMSO 213.765 MHz) *δ* (ppm): aglycone; 156.2 (C-2), 133.5 (C-3), 177.5 (C-4), 161.2 (C-5), 98.7 (C-6), 164.2 (C-7), 93.5 (C-8), 156.3 (C-9), 103.9 (C-10), 121.1 (C-1′), 116.0 (C-2′), 144.9 (C-3′), 148.5 (C-4′), 115.2 (C-5′), 122.0 (C-6′), sugar; 101.8 (C-1″), 71.2 (C-2″), 73.2 (C-3″), 67.9 (C-4″), 75.9 (C-5″), 60.1 (C-6″). The structure was confirmed by comparison with literature data [19,20,21].

*Quercetin 3-O-β-d-glucopyranoside* (**2**): Yellow amorphous powder (MeOH); UV/Vis *λ*_max_ nm 352, 256, 263 (sh); HRLC-MS *m*/*z* 465.0999 [M + H]^+^, ^1^H-NMR (*d*_6_-DMSO, 850.13 MHz) *δ* (ppm), 7.66 (1H, dd, *J* = 8.6, 2.3 Hz, H-6′), 7.53 (1H, d, *J* = 2.3 Hz, H-2′), 6.82 (1H, d, *J* = 8.6 Hz, H-5′), 6.41 (1H, d, *J* = 1.9 Hz, H-8), 6.20 (1H, d, *J* = 1.9 Hz, H-6), sugar; 5.46 (1H, d, *J* = 7.4 Hz, H-1″), 3.24 (1H, t like, *J* = 8.4 Hz H-2″), 3.22 (1H, t, *J* = 8.5 Hz, H-3″), 3.09 (1H, d, *J* = 5.7 Hz, H-4″), 3.08 (1H, m, H-5″), 3.32 (1H, td, *J* = 12.0, 6.0, 2.1 Hz, H-6a″), 3.58 (1H, d, *J* = 12.0 Hz, H-6b″). ^13^C-NMR (*d*_6_-DMSO 213.765 MHz) *δ* (ppm): aglycone; 156.2 (C-2), 133.3 (C-3), 177.4 (C-4), 161.2 (C-5), 98.7 (C-6), 164.2 (C-7), 93.5 (C-8), 156.3 (C-9), 104.0 (C-10), 121.2 (C-1′), 116.2 (C-2′), 144.8 (C-3′), 148.5 (C-4′), 115.2 (C-5′), 122.0 (C-6′), sugar; 100.8 (C-1″), 74.1 (C-2″), 76.5 (C-3″), 69.9 (C-4″), 77.6 (C-5″), 60.9 (C-6″). The structure was confirmed by comparison with literature data [19,21,22,58]. 

*Quercetin 3-O-β-d-(6″-*O*-malonyl)galactopyranoside* (**4**): Yellow amorphous powder (MeOH); UV/Vis *λ*_max_ nm 354, 256, 264 (sh); HRLC-MS *m*/*z* 551.1062 [M + H]^+^, ^1^H-NMR (*d*_6_-DMSO, 850.13 MHz) *δ* (ppm): aglycone; 7.67 (1H, dd, *J* = 8.3, 2.3 Hz, H-6′), 7.52 (1H, d, *J* = 2.2 Hz, H-2′), 6.81 (1H, d, *J* = 8.6 Hz, H-5′), 6.40 (1H, d, *J* = 2.0 Hz, H-8), 6.20 (1H, d, *J* = 2.0 Hz, H-6), sugar; 5.37 (1H, d, *J* = 8.1 Hz, H-1″), 3.57 (1H, m, H-2″), 3.36 (1H, dd, *J* = 8.9, 3.7 Hz, H-3″), 3.65 (1H, m, H-4″), 3.61 (1H, td, *J* = 6.2, 1.7 Hz, H-5″), 4.00 (1H, dd, *J* = 12.0, 5.8Hz, H-6a″), 4.20 (1H, dd, *J* =12.1, 2.3 Hz, H-6b″), acyl; 3.11 (2H, d, *J* = 16.0 Hz, H-2′′′). ^13^C-NMR (*d*_6_-DMSO 213.765 MHz) *δ* (ppm): aglycone; 156.2 (C-2), 133.4 (C-3), 177.1 (C-4), 161.2 (C-5), 98.6 (C-6), 164.1 (C-7), 93.4 (C-8), 156.3 (C-9), 103.8 (C-10), 121.5 (C-1′), 116.2 (C-2′), 144.7 (C-3′), 148.4 (C-4′), 115.1 (C-5′), 121.9 (C-6′), sugar; 101.7 (C-1″), 71.0 (C-2″), 73.1 (C-3″), 67.9 (C-4″), 72.4 (C-5″), 63.5 (C-6″), malonyl; 166.5 (C-1′′′), 41.0 (C-2′′′), 167.7 (C-3′′′). The structure was confirmed by comparison with literature data [23].

*Isorhamnetin 3-O-β-d-galactopyranoside* (**5**): Yellow amorphous powder (MeOH); UV/Vis *λ*_max_ nm 351, 254, 266 (sh); HRLC-MS *m*/*z* 479.1208 [M + H]^+^, ^1^H-NMR (*d*_4_-MeOD, 850.13 MHz) *δ* (ppm): aglycone; 7.59 (1H, dd, *J* = 8.5, 2.0 Hz, H-6′), 8.03 (1H, d, *J* = 2.0 Hz, H-2′), 6.90 (1H, d, *J* = 8.4 Hz, H-5′), 6.41 (1H, d, *J* = 1.9 Hz, H-8), 6.21 (1H, d, *J* = 1.9 Hz, H-6), 3.96 (3H, s, OCH_3_), sugar; 5.34 (1H, d, *J* = 7.4 Hz, H-1″), 3.82 (1H, dd, *J* = 9.6, 7.8 Hz, H-2″), 3.56 (1H, dd, *J* = 9.1, 2.8 Hz, H-3″), 3.84 (1H, dd, *J* = 3.2, 0.9 Hz, H-4″), 3.48 (1H, t, *J* = 8.5 Hz, H-5″), 3.47 (1H, td, *J* = 11.7, 5.7, 1.7 Hz, H-6a″), 3.65 (1H, dd, *J* = 11.8, 6.1 Hz, H-6b″). ^13^C-NMR (*d*_4_-MeOD, 213.765 MHz) *δ* (ppm): aglycone; 158.8 (C-2), 135.6 (C-3), 179.6 (C-4), 163.3 (C-5), 100.0 (C-6), 166.1 (C-7), 94.9 (C-8), 158.6 (C-9), 105.9 (C-10), 123.2 (C-1′), 114.7 (C-2′), 148.6 (C-3′), 151.0 (C-4′), 116.1 (C-5′), 123.8 (C-6′), 57.0 (OCH_3_), sugar; 104.5 (C-1″), 73.3 (C-2″), 75.2 (C-3″), 70.2 (C-4″), 77.4 (C-5″), 62.3 (C-6″). The structure was confirmed by comparison with literature data [24,25].

*Isorhamnetin 3-O-β-d-glucopyranoside* (**6**): Yellow amorphous powder (MeOH); UV/Vis *λ*_max_ nm 354, 254, 266 (sh); HRLC-MS *m*/*z* 479.1212 [M + H]^+^, ^1^H-NMR (*d*_4_-MeOD, 850.13 MHz) *δ* (ppm): aglycone; 7.58 (1H, dd, *J* = 8.4, 2.0 Hz, H-6′), 7.93 (1H, d, *J* = 2.0 Hz, H-2′), 6.91 (1H, d, *J* = 8.3 Hz, H-5′), 6.41 (1H, d, *J* = 1.9 Hz, H-8), 6.21 (1H, d, *J* = 1.9 Hz, H-6), 3.95 (3H, s, OCH_3_), sugar; 5.41 (1H, d, *J* = 7.8 Hz, H-1″), 3.46 (1H, t like, *J* = 8.6 Hz, H-2″), 3.45 (1H, dd, *J* = 9.4, 8.2 Hz, H-3″), 3.30 (1H, m, *J* = 9.4 Hz, H-4″), 3.24 (1H, m, H-5″), 3.57 (1H, dd, *J* = 11.9, 5.8 Hz, H-6a″), 3.73 (1H, dd, *J* = 12.0, 2.5 Hz, H-6b″). ^13^C-NMR (*d*_4_-MeOD, 213.765 MHz) *δ* (ppm): aglycone; 158.8 (C-2), 135.5 (C-3), 179.6 (C-4), 163.3 (C-5), 100.0 (C-6), 166.1 (C-7), 94.9 (C-8), 158.6 (C-9), 105.9 (C-10), 123.2 (C-1′), 114.6 (C-2′), 148.6 (C-3′), 151.0 (C-4′), 116.1 (C-5′), 122.5 (C-6′), 57.0 (OCH_3_), sugar; 103.7 (C-1″), 76.1 (C-2″), 78.2 (C-3″), 71.6 (C-4″), 78.7 (C-5″), 62.7 (C-6″). The structure was confirmed by comparison with literature data [22,25,26].

*Isorhamnetin 3-O-β-d-(6″-*O*-malonyl)galactopyranoside* (**7**): Yellow amorphous powder (MeOH); UV/Vis *λ*_max_ nm 350, 254, 266 (sh); HRLC-MS *m*/*z* 565.1216 [M + H]^+^, ^1^H-NMR (*d*_4_-MeOD, 850.13 MHz) *δ* (ppm): aglycone; 7.62 (1H, dd, *J* = 8.3, 2.1 Hz, H-6′), 7.90 (1H, d, *J* = 2.0 Hz, H-2′), 6.92 (1H, d, *J* = 8.4 Hz, H-5′), 6.44 (1H, d, *J* = 2.1 Hz, H-8), 6.23 (1H, d, *J* = 2.1 Hz, H-6), 3.97 (3H, s, OCH_3_), sugar; 5.21 (1H, d, *J* = 7.6 Hz, H-1″), 3.81 (1H, m, H-2″), 3.58 (1H, t, *J* = 9.7 Hz, H-3″), 3.89 (1H, d, *J* = 4.3 Hz, H-4″), 3.86 (1H, t, *J* = 9.1 Hz, H-5″), 4.29 (1H, dd, *J* = 11.4, 4.4 Hz, H-6a″), 4.49 (1H, dd, *J* = 11.6, 8.4 Hz, H-6b″). ^13^C- NMR (*d*_4_-MeOD, 213.765 MHz) *δ* (ppm): aglycone; 157.6 (C-2), 134.0 (C-3), 178.0 (C-4), 161.7 (C-5), 98.5 (C-6), 164.6 (C-7), 93.6 (C-8), 157.0 (C-9), 104.3 (C-10), 121.5 (C-1′), 113.0 (C-2′), 146.9 (C-3′), 149.5 (C-4′), 114.7 (C-5′), 122.4 (C-6′), 55.4 (OCH_3_), sugar; 103.3 (C-1″), 71.4 (C-2″), 73.4 (C-3″), 69.0 (C-4″), 73.4 (C-5″), 63.1 (C-6″), acyl; 166.3 (C-1′′′). The structure was confirmed by comparison with literature data [23,27].

*Isorhamnetin 3-O-β-d-(6″-*O*-malonyl)glucopyranoside* (**8**): Yellow amorphous powder (MeOH); UV/Vis *λ*_max_ nm 355, 254, 266 (sh); HRLC-MS *m*/*z* 565.1208 [M + H]^+^, ^1^H-NMR (*d*_4_-MeOD, 850.13 MHz) *δ* (ppm): aglycone; 7.61 (1H, dd, *J* = 8.5, 2.0 Hz, H-6′), 7.88 (1H, d, *J* = 2.1 Hz, H-2′), 6.91 (1H, d, *J* = 8.4 Hz, H-5′), 6.44 (1H, d, *J* = 2.1 Hz, H-8), 6.22 (1H, d, *J* = 2.1 Hz, H-6), 3.95 (3H, s, OCH_3_), sugar; 5.22 (1H, d, *J* = 7.6 Hz, H-1″), 3.40 (1H, m, *J* = 8.6 H-2″), 3.43 (1H, t, *J* = 8.7 Hz, H-3″), 3.35 (1H, t, *J* = 9.7 Hz, H-4″), 3.47 (1H, t, *J* = 8.5 Hz, H-5″), 4.19 (1H, dd, *J* = 12.0, 5.6 Hz, H-6a″), 4.23 (1H, dd, *J* = 12.0, 2.3 Hz, H-6b″). ^13^C-NMR (*d*_4_-MeOD, 213.765 MHz) *δ* (ppm): aglycone; 157.7 (C-2), 135.6 (C-3), 173.9 (C-4), 161.7 (C-5), 98.5 (C-6), 164.6 (C-7), 93.6 (C-8), 157.1 (C-9), 104.3 (C-10), 121.6 (C-1′), 113.0 (C-2′), 147.0 (C-3′), 149.6 (C-4′), 114.6 (C-5′), 122.7 (C-6′), 55.4 (OCH_3_), sugar; 103.1 (C-1″), 74.2 (C-2″), 76.5 (C-3″), 69.9 (C-4″), 74.4 (C-5″), 63.4 (C-6″), acyl; 169.0 (C-1′′′). The structure was confirmed by comparison with literature data [27]. 

*2,3-O-Dicaffeoyltartaric acid* (**CA**): White amorphous powder (MeOH); UV/Vis *λ*_max_ nm 331, 302 (sh), 245; HRLC-MS *m*/*z* 497.0681 [M + Na]^+^, ^1^H-NMR (*d*_6_-DMSO, 850.13 MHz) *δ* (ppm): 5.68 (2H, s, H-2, H-3), 7.10 (2H, d, *J* = 2.1 Hz, H-2′, H-2″), 6.78 (2H, d, *J* = 8.1 Hz, H-5′, H-5″), 7.08 (2H, dd, *J* = 8.2, 2.1 Hz, H-6′, H-6″), 7.56 (1H, d, *J* = 15.8 Hz, H-7′, H-7″), 6.36 (1H, d, *J* = 15.8 Hz, H-8′, H-8″). ^13^C-NMR (*d*_6_-DMSO, 213.765 MHz) *δ* (ppm): 167.6 (C-1, C-4), 70.7 (C-2, C-3), 125.2 (C-1′, C-1″), 115.3 (C-2′, C-2″), 145.6 (C-3′, C-3″), 148.9 (C-4′, C-4″), 115.8 (C-5′, C-5″), 121.7 (C-6′, C-6″), 147.0 (C-7′, C-7″), 112.3 (C-8′, C-8″), 165.5 (C-9′, C-9″). The structure was confirmed by comparison with literature data [28].

### 3.4. Quantitative Determination 

Leaves of *R. cirrhosa* and *R. maritima* were cut into small pieces and extracted with 50% aqueous methanol, the flavonoid content of the extract was characterized by analytical HPLC with DAD and HR–LCMS. Quantitative determination: 10–40 mg of dried plant material was weighed and extracted with 3–5 mL of 50% aqueous methanol for 2 hours at room temperature. Four replicate samples were made. Prior to injection, the solutions were filtered through a 0.45 μm Millipore membrane filter. HPLC calibration curves of quercetin 3-*O-β-*d-glucopyranoside (≥90% (HPLC), Sigma-Aldrich, Sigma-Aldric, St. Louis, MO, USA) and caffeic acid (≥98% (HPLC), Sigma-Aldrich) were used to determine the quantitative amounts of flavonoids and phenolic compounds, respectively. The results are presented as milligrams quercetin 3-*O-β-*d-glucopyranoside *or* caffeic acid equivalents ± one standard deviation (SD) per gram of dry weight (DW) plant material. Two sample t-test assuming unequal variances with a *p*-value of 0.05 was used to determine if the means of two different measurements were equal or not. Standard error bars were calculated using the STDEV. P function in excel, and represent one standard deviation (*n* = 4 or number of replicates).

### 3.5. Method Validation 

The established HPLC method was validated for linearity, sensitivity, precision and accuracy, as previously described [51]. LOD and LOQ were calculated based on standard deviation of y-intercepts of the regression line (SD) and the slope (S), using the equations LOD = 3.3 × SD/S and LOQ = 10 × SD/S. Recovery study was performed in triplicate by adding known amounts of quercetin 3-*O-β-*d-glucopyranoside to crude extracts of *R. cirrhosa*. Data for calibration curves, test ranges, limit of detection (LOD) and limit of quantification (LOQ) for quercetin 3-*O-β-*d-glucopyranoside (90%, Sigma-Aldrich Sigma) and caffeic acid (Sigma-Aldrich) are presented in Table 5. The recovery was ranging from 93.3% to 94.8% for quercetin 3-*O-β-*d-glucopyranoside with a mean of 94.0 ± 2.0% (Table 5).

### 3.6. DPPH Radical Scavenging 

The stable 1,1-diphenyl-2-picryl hydrazyl radical (DPPH·) was used for determination of free radical-scavenging activity of *R. cirrhosa* extracts and isolated mixtures of flavonoids (purity ≥ 75% (HPLC)). Different sample concentrations of the extracts were prepared, and 0.05 mL of each sample was added to a 2.95 mL methanolic solution of DPPH· (45 μg/mL). A UV-1800 UV spectrophotometer (Shimadzu Scientific Instruments, Columbia, MD, USA) was used for the antioxidant assays. The UV/Vis absorbance at 517 nm was measured every 30 s for 5 min. The experiment was repeated three times, and the results are presented as mean ± standard deviation (*n* = 3). Trolox (97%, Sigma-Aldrich), chicoric acid (≥95% (HPLC), Sigma-Aldrich), quercetin (≥95% (HPLC), Sigma-Aldrich), quercetin 3-*O*-*β*-d-glucopyranoside (≥90% (HPLC), Sigma-Aldrich) and rutin (≥95% (HPLC), Sigma-Aldrich) were used as standard controls. Percent radical-scavenging was calculated as 100 × (A_start_ − A_end_)/(A_start_), where A_start_ is the absorbance before addition of the sample, and A_end_ is the absorbance value after 5 min of reaction time. Percent scavenging IC_50_ values were calculated from a linear regression plot of percent scavenging (%) against logarithmic concentration of the test compound [59]. IC_50_ values denote the concentration of sample which is required to scavenge 50% of DPPH· free radicals.

## 4. Conclusions

In this study, the polyphenolic content of *Ruppia cirrhosa* and *Ruppia marittima* was characterized for the first time using NMR-spectroscopy, HRLC-MS and HPLC-UV. Both *Ruppia* species contained high amounts of chicoric acid (10.6–30.2 mg/g DW), followed by relatively high amounts of flavonoid glycosides (5.9–14.7 mg/g DW). The eight flavonoids identified were based on quercetin and isorhamnetin with 3-*O*-galactopyranosides or 3-*O*-glucopyranosides, four of these were malonylated. This is the first report of 3-*O*-galactopyranosides and malonylated flavonoids of quercetin and isorhamnetin isolated from aquatic plants. The seasonal variations of flavonoids and phenolics were examined by analyzing *R. cirrhosa* samples in October, March and August. Highest flavonoid content was found in August, whereas the highest concentration of chicoric acid was observed in March.

Extracts of *R. cirrhosa* showed low to moderate DPPH· antioxidant activity, however, partially purified extract and isolated compounds showed strong to very strong antioxidant activities, with IC_50_ values ranging from 12.1 to 88.4 μg/mL.

## Figures and Tables

**Table 1:** IC_50_ values of extract of *Ruppia cirrhosa* and isolated compounds from *R. cirrhosa*.

Extracts and Compounds	DPPH· ^1^ IC_50_ (μg/mL)
*R. cirrhosa* crude extract (October)	175.7 ± 7.8
*R. cirrhosa* crude extract (August)	152.9 ± 8.1
*R. cirrhosa* purified extract	31.8 ± 0.7
**3 + 4**	12.1 ± 2.2
**5 + 6**	88.4 ±7.0
**7 + 8**	51.7 ± 6.8
**CA**	23.0 ± 3.2

^1^ IC_50_ values calculated by linear regression of % scavenging and logarithmic concentration.

**Table 2:** IC_50_ values of reference standards.

Reference Standard	DPPH· ^1^ IC_50_ (μg/mL)
quercetin (≥95%)	5.5 ± 0.3
quercetin 3-*O-β-*d-glucopyranoside (≥90%)	11.0 ± 1.0
rutin (≥95%)	13.9 ± 0.7
Trolox (≥97%)	6.1 ± 0.4
chicoric acid (≥95%)	9.7 ± 1.7

^1^ IC_50_ values calculated by linear regression of % scavenging and logarithmic concentration.

**Table 3:** Quantitative amounts of individual flavonoids and phenolic acids in leaves of *Ruppia cirrhosa* (*R. cirr*.) and *Ruppia maritima* (*R. mar*.) collected in summer 2017 from five localities (A–E). ^1,2^

Compound	*R. cirr*. (A) (mg/g)	*R. cirr*. (B) (mg/g)	*R. cirr*. (C) (mg/g)	*R. mar*. (D) (mg/g)	*R. mar*. (E) (mg/g)
**CA**	12.7 ± 2.5 ^a^	11.9 ± 2.2 ^a^	11.1 ± 1.4 ^a^	30.2 ± 4.3 ^b^	27.9 ± 5.1 ^b^
**1**	2.2 ± 0.3 ^d^	0.7 ± 0.1	1.1 ± 0.1 ^g^	2.0 ± 0.5 ^d^	1.1 ± 0.2 ^g^
**2**	1.3 ± 0.2	0.5 ± 0.04 ^e^	1.0 ± 0.1 ^f^	1.0 ± 0.2 ^f^	0.6 ± 0.1 ^e^
**3**	0.9 ± 0.1	0.4 ± 0.04 ^e^	0.7 ± 0.04 ^f,g^	0.6 ± 0.1 ^b,f^	0.6 ± 0.1 ^b,e,g^
**4**	1.9 ± 0.3	0.7 ± 0.05 ^a^	0.8 ± 0.04 ^a^	1.5 ± 0.3 ^b^	1.4 ± 0.2 ^b^
**5**	2.9 ± 0.4	1.0 ± 0.1 ^a^	1.0 ± 0.1 ^a^	1.6 ± 0.3 ^b^	2.0 ± 0.2 ^b^
**6**	2.1 ± 0.2 ^d^	0.8 ± 0.1	1.3 ± 0.1 ^f^	1.7 ± 0.3 ^b,d,f^	1.6 ± 0.2 ^b^
**7**	1.1 ± 0.2 ^c^	0.6 ± 0.1 ^a,e^	0.5 ± 0.04 ^a,f^	0.6 ± 0.07 ^e,f^	1.1 ± 0.2 ^c^
**8**	2.2 ± 0.3 ^c^	1.1 ± 0.1	1.5 ± 0.1	1.8 ± 0.2	2.3 ± 0.3 ^c^
sum flavonoids	14.7 ± 1.9	5.9 ± 0.5	7.9 ± 0.5	10.7 ± 1.7 ^b^	10.7 ± 1.5 ^b^
sum phenolics	27.4 ± 4.3	17.7 ± 2.1 ^a^	19.0 ± 1.8 ^a^	41.0 ± 5.7 ^b^	38.5 ± 6.3 ^b^

^1^ Amounts are expressed in mg/g (mean value ± SD, *n* = 4) dry weigth, based on quercetin 3-*O*-β-d-glucopyranoside (flavonoids) or caffeic acid (chicoric acid) equivalents.^2^ same letters (a–g) indicate where values are significantly *not* different, *p* > 0.05 with a *t* test.

**Table 4:** Quantitative amounts of individual flavonoids and chicoric acid in leaves of *Ruppia cirrhosa* collected in October 2016, March 2017 and August 2017. ^1,2^

Compound	16 October (mg/g)	17 March (mg/g)	17 August(mg/g)
**CA**	10.6 ± 2.5 ^a^	29.2 ± 6.3	12.7 ± 2.5 ^a^
**1**	0.8 ± 0.1	2.2 ± 0.4 ^b^	2.2 ± 0.3 ^b^
**2**	0.6 ± 0.1	0.8 ± 0.2	1.3 ± 0.2
**3**	0.7 ± 0.1 ^a^	0.9 ± 0.2 ^a^	0.9 ± 0.1 ^a^
**4**	1.1 ± 0.2	2.5 ± 0.6 ^b^	1.9 ± 0.3 ^b^
**5**	1.1 ± 0.2	1.5 ± 0.3	2.9 ± 0.4
**6**	0.8 ± 0.1 ^a^	0.7 ± 0.1 ^a^	2.1 ± 0.2
**7**	1.2 ± 0.2 ^a^	1.1 ± 0.2 ^a^	1.1 ± 0.2 ^a^
**8**	2.0 ± 0.3	1.4 ± 0.3	2.2 ± 0.3
sum flavonoids	8.4 ± 1.1 ^a^	11.1 ± 2.4 ^a^	14.7 ± 1.9
sum phenolics	19.0 ± 3.0	40.3 ± 8.7	27.4 ± 4.3

^1^ Amounts are expressed in mg/g (mean value ± SD, *n* = 4) dry weight, based on quercetin 3-*O*-*β*-d-glucopyranoside (flavonoids) or caffeic acid (chicoric acid) equivalents. ^2^ same letters (a,b) indicate where values are significantly *not* different, *p* > 0.05 with a *t* test.

**Table 5:** Calibration curve, LOD and LOQ for quercetin 3-*O-β-*d-glucopyranoside (≥90%, Sigma Aldrich) and caffeic acid (≥98%, Sigma-Aldrich).

	Calibration Curve (μg/mL)	*R*^2^	Test Range (μg/mL)	LOD (μg/mL)	LOQ (μg/mL)	Spike Recovery %
quercetin 3-*O-β-*d-glucopyranoside	*y* = 36.56*x* − 11.8	0.9998	2.5–80	2.0	6.0	94.0 ± 2.0
caffeic acid	*y* = 102.8*x* + 12.8	0.9994	10–80	1.1	3.3	
